# The Retinome – Defining a reference transcriptome of the adult mammalian retina/retinal pigment epithelium

**DOI:** 10.1186/1471-2164-5-50

**Published:** 2004-07-29

**Authors:** Heidi L Schulz, Thomas Goetz, Juergen Kaschkoetoe, Bernhard HF Weber

**Affiliations:** 1 Institute of Human Genetics, Biocenter, University of Wuerzburg, D-97074 Wuerzburg, Germany; 2 German Cancer Research Center, Central Spectroscopic Department, D-69120 Heidelberg, Germany

## Abstract

**Background:**

The mammalian retina is a valuable model system to study neuronal biology in health and disease. To obtain insight into intrinsic processes of the retina, great efforts are directed towards the identification and characterization of transcripts with functional relevance to this tissue.

**Results:**

With the goal to assemble a first genome-wide reference transcriptome of the adult mammalian retina, referred to as the retinome, we have extracted 13,037 non-redundant annotated genes from nearly 500,000 published datasets on redundant retina/retinal pigment epithelium (RPE) transcripts. The data were generated from 27 independent studies employing a wide range of molecular and biocomputational approaches. Comparison to known retina-/RPE-specific pathways and established retinal gene networks suggest that the reference retinome may represent up to 90% of the retinal transcripts. We show that the distribution of retinal genes along the chromosomes is not random but exhibits a higher order organization closely following the previously observed clustering of genes with increased expression.

**Conclusion:**

The genome wide retinome map offers a rational basis for selecting suggestive candidate genes for hereditary as well as complex retinal diseases facilitating elaborate studies into normal and pathological pathways. To make this unique resource freely available we have built a database providing a query interface to the reference retinome [1].

## Background

The mammalian retina is a highly structured tissue developmentally originating from neuroectodermal evagination of the diencephalon and subsequent invagination processes resulting in the formation of two cellular layers which ultimately give rise to the inner neural retina and the outer retinal pigment epithelium (RPE) monolayer [2]. In the adult, the neural retina consists of approximately 55 distinct cell types histologically structured into three layers of cells (photoreceptors, intermediate neurons and ganglion cells) and two layers of neuronal interconnections (outer and inner plexiform layers) [3]. The RPE is differentiated into polarized cells with an apical and a basal orientation separating the neural retina from the underlying choroidal blood supply. With its apical microvilli-like processes, the RPE establishes an intimate contact with the photoreceptor outer segments to sustain their metabolic support and maintain photoreceptor integrity [4]. Together, the neural retina and the RPE provide the structural and functional basis for light perception by ensuring the capture of photons, the conversion of light stimuli into complex patterns of neuronal impulses and the transmission of the initially processed signals to the higher visual centers of the brain.

Recent progress in retinal research has greatly enhanced our current understanding of basic functional processes in the adult retina (e.g. [4,5]). A great deal of effort has focused on the molecular dissection of the phototransduction pathway and the retinoid cycle (e.g. ref. [6]). Besides elucidating physiological mechanisms in normal tissue, the identification of genes involved in hereditary retinal disease has provided another valuable source of insight into functional pathways of the retina and the RPE (reviewed in [7,8]).

Despite these advances, a remaining challenge is to obtain a reference genome-wide expression map of the retina/RPE transcriptome, further facilitating the identification of retinal susceptibility genes, but most importantly, offering an invaluable resource for functional genomics studies. Initial analyses of human [9,10] and mouse [11] whole genome sequences and the use of more recent comparative gene prediction algorithms [12,13] suggest an overall number of mammalian gene loci in the range of 35,000 to 45,000. These estimates have largely been validated by experimental data on gene transcription [14,15] although alternative promoter usage, differential exon splicing during mRNA maturation, alternative usage of polyadenylation sites and other post-transcriptional modifications may further increase the genetic diversity required to encode the full complement of cellular transcripts [16,17]. In addition, there may be a considerable number of non-coding genes unaccounted for by current annotations [18].

In recent years, a number of approaches and technologies were adopted to identify genes expressed in the retina/RPE of human, cow, dog and mouse including data-mining and assembly of publically available expressed sequence tag (EST) information [19-23], sequencing of cDNA libraries generated via conventional methods [24-29] or via normalization techniques [30,31], hybridization to gene arrays of various formats [32,33] and serial analysis of gene expression (SAGE) [34,35]. Suppression subtractive hybridization (SSH) has been shown to be an efficient technique with which differentially expressed genes can be normalized and enriched over 1000-fold in a single round of hybridization [36]. Subsequently, applications of SSH to identify retina and RPE-enriched genes have been reported [37-39].

Based on a comprehensive survey of data available from 27 independent studies applying a wide spectrum of gene identification approaches we have now assembled a first genome-wide reference transcriptome of the adult mammalian retina/RPE. This reference transcriptome comprises 13,037 non-redundant transcripts and likely reflects up to 90% of the mammalian retinome.

## Results

A total of 481,137 primary datasets on gene transcripts from the adult mammalian retina/RPE tissues have been generated in 27 independent studies (Table 1). Of these, 52,630 datasets (31,814 from retina, 11,632 from RPE and 9,184 from retina/RPE) were available and attributable to unique LocusLink identifiers (IDs). Correcting for gene redundancy within and between studies yielded a catalogue of 15,645 retinal/RPE genes. A survey of incidence and origin of each of these genes in the various studies analyzed demonstrated that 2,608 transcripts were found only once (see additional File 1) while the remaining 13,037 genes (see additional File  2) were confirmed in at least two and up to 16 independent gene identification approaches (Table 2). Thus, the latter compilation of genes may represent a more conservative description of the retinome minimizing a potential bias in data ascertainment. Of the 13K retinome, 1,411 genes were solely identified in retinal studies (see additional File 3) while 246 genes were exclusively found in the RPE datasets (see additional File 4).

**Table 1:** Studies identifying adult mammalian retina / RPE transcripts and details on gene data retrieval

**Reference**	**Source / method ^a)^**	**Species ^b)^**	**Original dataset**	**No. of genes retrieved ^c) ^(non-redundant)**	**No. of genes identified in ≥ 2 studies**
	***In-silico *projects**				
[20]	Retina, TIGR (ID: version 3.3)	Hs	1,047	30	30
[19]	Retina, TIGR (ID: version 3.3)	Hs	1,315	11	11
[21]	Retina, UniGene (ID: build 118)	Hs	4,974	1,485	1,480
[22]	Retina, UniGene (ID: build 113)	Hs	6,190	46	45
[23]	Retina, EST (dbEST)	Hs	40,000	117	110
UniGene (Build 162) ^d)^	Retina	Hs	3,560	1,612	1,211
UniGene (Build 162) ^d)^	RPE	Hs	1,760	1,506	1,116
UniGene (Build 162) ^d)^	Retina & RPE	Hs	11,976	9,178	9,178
	**cDNA library sequencing**				
[24]	Fovea, conventional	Hs	209	40	38
[25]	Retina, conventional	Hs	607	475	465
[37]	Retina & RPE, SSH	Hs	401	6	6
[30]	Retina, subtracted	Hs	137	49	48
[31]	Retina, PC and subtracted	Cf	173	66	65
[26]	RPE, primary and amplified, conventional	Hs	2,101	336	330
[38]	RPE, SSH	Bt	1,000	35	34
[27] and online ^e)^	Retina, PC	Hs	2,701	2,096	2,059
[28] and online ^f)^	RPE, PC	Hs	6,182	3,657	3,608
[29]	Retina, PC (ID: MRA)	Mm	1,793	421	412
[39]	Retina, SSH	Hs	1,080	310	301
[39]	RPE, SSH	Bt	2,350	343	329
	**Microarray Analysis**				
[32]	Retina, Affymetrix (ID: Mu11K subB)	Mm	6,540	67	67
[33]	Retina, custom array	Hs	10,034	530	508
	**Serial Analysis of Gene Expression**				
[34]	Retina, adult	Mm	54,009	4,233	3,974
[35]	Retina (ID: HMAC2)	Hs	102,359	7,269	6,919
[35]	Retina (ID: HPR1)	Hs	59,661	5,689	5,512
[35]	Retina (ID: HPR2)	Hs	105,312	7,268	6,955
[35]	RPE (ID: HRPE1)	Hs	53,666	5,755	5,211
**Total (with inter-study redundancy)**				**52,630**	**50,022**
**Total (without redundancy)**				**15,645**	**13,037**

^a) ^TIGR: The Institute for Genomic Research; EST: expressed sequence tag; RPE: retinal pigment epithelium; SSH: suppression subtractive hybridization; PC: primary, conventional ^b) ^Hs: Homo sapiens; Mm: Mus musculus; Cf: Canis familiaris; Bt: Bos taurus ^c) ^Genes with LocusLink ID identified based on publically available information ^d) ^[67], For query terms see (see additional File 15 ^e) ^[68], March 2003 ^f) ^[69], March 2003

**Table 2:** Frequency of unique genes in studies

**No. of studies**	**No. of unique genes**
1	2,608 ^a)^
2	4,162
3	2,368
4	2,002
5	1,736
6	1,282
7	768
8	385
9	178
10	84
11	37
12	23
13	6
14	2
16	4
**Total**	**15,645**

^a) ^not included in assembly of 13K retinome (see additional File 1)

To assess the degree of completeness of the adult mammalian retinome, we compared the LocusLink IDs of the 13,037 transcripts to partial lists of genes known i) to be specifically expressed in the retina/RPE (category I, n = 43) (see additional File 5), ii) to play a role in the phototransduction pathway/vitamin A cycle (category II, n = 57) (see additional File 6), iii) to encode retinal/RPE proteins verified by immunohistochemistry (category III, n = 260) (see additional File 7), and iv) to be associated with syndromic and non-syndromic retinal disease (category IV, n = 102) (see additional File 8). The data show that the compiled retinome covers all retina/RPE-specific transcripts (43/43) and 53/57 (93%) of the phototransduction pathway/vitamin A cycle genes. Known retinal/RPE proteins are represented by 204/260 (79%) transcripts while 87/102 (85%) genes known to be involved in retinal diseases are found in the 13K retinome collection (Table 3). To further evaluate the significance of these findings, partial transcriptomes of heart (n = 3,660; see additional File 9), liver (n = 5,780; see additional File 10) and prostate (n = 7,018; see additional File 11) were assembled and compared to the four selected categories. In category 1, none of the retina/RPE-specific genes were present in the heart, liver, or prostate partial transcriptomes while category 2 revealed four of 16 expected (25%, heart), 7 of 25 (28%, liver) and 9 of 31 (29%, prostate) genes. In category 3, 59/73 (81%, heart), 82/115 (71%, liver) and 92/140 (66%, prostate) genes were present. Retinal disease genes (category 4) were found at a rate of 18 of 29 (62%, heart), 26 of 45 (58%, liver) and 28 of 55 (51%, prostate) (Table 3). The expected values for the partial transcriptomes were calculated by adjusting the respective transcriptome sizes relative to the total number of transcripts of the retinome.

**Table 3:** Representation of retinome and partial assemblies of heart, liver, and prostate transcriptomes in defined retina/RPE gene groups

**Transcriptome**	**No. of non-redundant genes identified in ≥ 2 studies ^a)^**	**Retina / RPE-specific genes ^b) ^(n = 43)**	**Vitamin A / phototrans-duction pathway ^c) ^(n = 57)**	**Retina / RPE genes (verified by immunohisto-chemistry ^d)^) (n = 260)**	**Retinal disease genes ^e) ^(n = 102)**	**Source / Reference**
Retinome	13,037	43	53	204	87	Present publication
Heart (partial)	3,660	0	4	59	18	UniGene Build 166, SAGE library GSM1499
Liver (partial)	5,780	0	7	82	26	UniGene Build 166, SAGE library GSM785
Prostate (partial)	7,018	0	9	92	28	UniGene Build 166, SAGE libraries GSM685, GSM739, GSM764

^a) ^See additional Files 2, 9, 10 and 11 ^b) ^See additional File 5 ^c)^ See additional File 6^d) ^See additional File 7 ^e) ^See additional File 8

A comparison of the 13K retinome with partial transcriptomes of heart, liver, and prostate suggests a high degree of overlapping expression between retina/RPE and heart (3,496/3,660), liver (5,343/5,780) and prostate (6,471/7,018). A total of 2,330 genes are expressed in all tissues and represent putative "housekeeping" genes (see additional File 12). It should be noted that the low number of ubiquitously expressed genes is largely due to the fragmentary nature of the heart, liver, and prostate transcriptomes. With increasing transcriptome complexities this number is likely to increase. Analysis of the least complete transcriptome, the heart, reveals that 2,330/3,660 (64%) transcripts can be classified as ubiquitously expressed (see additional Files 9 and 12) while a maximum of 1,330/3,660 (36%) genes may display tissue-restricted or tissue-specific expression. A comparison of more complete transcriptomes may significantly reduce the latter estimate. So far 5,051 genes are only found in the retinome representing a collection of "retinome-enriched" transcripts, while 7,986 are also present in at least one of the partial transcriptomes of the heart, liver or prostate. Thirty-two genes were found to be expressed in heart, liver and prostate but not in the retinome (see additional File 13).

**Figure 1:**
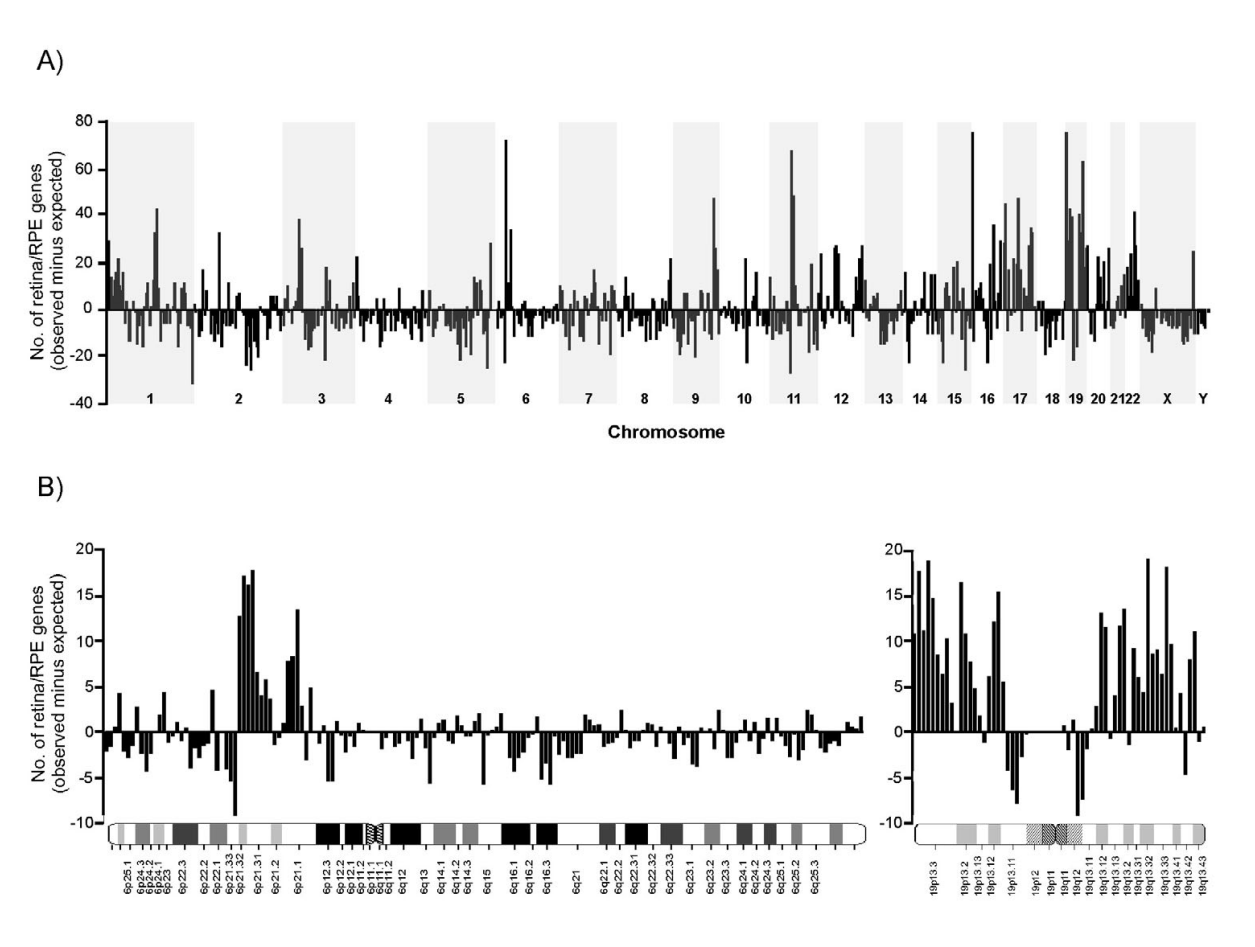
**Chromosomal distribution of transcripts defining the reference retinome. **(**A**) The distribution of 13,037 retinome genes over the human genome is shown as the difference between the number of observed and expected transcripts in window sizes of 5 Mb along the chromosomes (abscissa). The number of expected genes was based on 43,109 SGP-predicted transcripts. To correct for gene density per bin, the SGPs were adjusted by a factor of 0.30 (13,037/43,109). Positive/negative ordinate values indicate regions of enrichment/depletion of retinome-encoded transcripts. (**B**) Close-up of chromosomes 6 and 19 calculated for a window size of 1 Mb along the two chromosomes.

The distribution of the mammalian retinome across the human genome was assessed by a paired comparison of the number of reference retinome genes (13,037) versus the number of human non-redundant syntenic gene predictions (SGPs) (43,109) [40] along 618 five-Mb windows (Fig. 1a). Correction for the total number of SGPs relative to the number of retinome genes positioned on average 21.1 (median 19.9) SGPs per window compared to 21.3 (median 15.0) retinal genes per window. Based on the Wilcoxon two-sample paired signed rank test, the null hypothesis assuming similar distribution of the SGPs and the retinome genes across the five-Mb windows can be rejected at p < 0.01. While their overall distribution greatly parallels that of the SGPs, retinome genes tend to cluster in several chromosomal regions most prominently on chromosomes 6p22.1-p21.31, 11q12.2-q13.1, 16p13.3, 19p13.3 and 19q13.32-q13.33 (Fig. 1a,1b).

To provide positional candidates for syndromic and non-syndromic hereditary retinopathies, the 13K reference retinome as well as the "retinome-enriched" transcripts (5,051 transcripts) were superimposed onto the disease intervals of 42 thus far uncloned retinal disorders (Table 4). In many instances, this results in a significant reduction of genes in the respective intervals offering a manageable number of candidates for retinal diseases (e.g. the *RP29 *locus contains 28 SGPs of which 5 are present in the reference retinome including *GPM6A*, *WDR17*, *FLJ22649*, *VEGFC*, *AGA*). The number of possible candidates is further reduced in the "retinome-enriched" transcript category to *GPM6A *and *VEGFC*. To make the information on the reference retinome available, we have created the interactive RetinaCentral database, a research portal which collects and stores information on genes and proteins functionally relevant to the retinal tissues [1]. We have implemented an interactive data retrieval system that presently contains linked information on the 13,037 genes of the 13K reference retinome. Database scripts were programmed to synchronize the data with LocusLink index files [41] which are updated daily [42].

**Table 4:** Number of genes mapped to retinal disease loci

**Disease**	**Chr.**	**Flanking DNA marker**		**Size of locus (Mb)**	**No. of SGPs (n = 43,109)**	**No. of retinome transcripts ^a) ^(n = 13,307)**	**No. of "retinome-enriched" transcripts (n = 5,051)**
*AA*	11	*D11S1323*	*D11S902*	11.21	213	65	13
*AIED*	X	*DXS7*	*DXS72*	37.33	479	129	49
*AXPC1*	1	*D1S2692*	*D1S414*	3.00	43	7	3
*BBS3*	3	*D3S1603*	*D3S1271*	2.28	28	10	1
*BBS5*	2	*D2S142*	*D2S326*	16.81	353	50	24
*BCD*	4	*D4S3051*	*D4S1652*	2.06	17	2	1
*CACD*	17	*D17S1810*	*CHLC.GAT7B03*	3.17	118	76	36
*COD2*	X	*DXS292*	*DXS1113*	6.1	37	5	3
*COD4*	X	*DXS10042*	*DXS8060*	32.44	436	117	45
*CORD1*	18	marker position not available		-	-	-	-
*CORD4*	17	marker position not available		-	-	-	-
*CORD8*	1	*D1S442*	*D1S2681*	19.86	485	222	74
*CORD9*	8	*D8S1820*	*D8S532*	12.79	179	43	16
*CRV*	3	*D3S3564*	*D3S1578*	11.2	271	157	72
*CYMD*	7	*D7S435*	*D7S526*	1.56	25	13	7
*EVR3*	11	*GATA34E08*	*D11S4102*	14.00	192	35	13
*LCA3*	14	*D14S606*	*D14S610*	5.95	178	36	15
*LCA5*	6	*D6S1551*	*D6S1694*	36.08	541	263	93
*LCA9*	1	*D1S1612*	*D1S228*	5.52	130	51	22
*MCDR1*	6	*D6S249*	*D6S1671*	2.44	45	2	0
*MCDR2*	4	*D4S3023*	*D4S3022*	21.00	253	57	20
*MCDR3*	5	*D5S1981*	*D5S2031*	19.94	184	36	14
*MCDR4*	14	*D14S261*	-	10.00 ^b)^	58	11	4
*MRST*	15	*D15S211*	*D15S152*	4.70	106	24	11
*OPA2*	X	*DXS993*	*DXS991*	14.31	245	69	24
*OPA4*	18	*D18S34*	*D18S548*	2.55	23	3	1
*PRD*	X	*MAOB*	*DXS426*	3.92	67	18	6
*RCD1*	6	marker position not available		-	-	-	-
*RNANC*	10	*D10S1225*	*D10S1418*	5.76	58	9	2
*ROA1*	8	*D8S1702*	*D8S1794*	11.87	150	30	13
*RP6*	X	*DXS28*	*DXS164*	4.92	60	4	2
*RP17*	17	*D17S1607*	*D17S948*	7.18	130	44	17
*RP22*	16	*D16S287*	*D16S420*	6.33	92	40	14
*RP23*	X	*DXS1223*	*DXS7161*	10.51	140	30	15
*RP24*	X	*DXS8094*	*DXS8043*	7.75	74	6	3
*RP28*	2	*D1S1337*	*D2S286*	45.78	674	194	68
*RP29*	4	*D4S3035*	*D4S2417*	3.66	28	5	2
*STGD4*	4	*D4S1582*	*D4S2397*	16.56	187	33	10
*USH1A*	14	*D14S99E*	*D14S292*	6.98	134	44	18
*USH1E*	21	*D21S1905*	*D21S1913*	11.48	77	19	10
*USH2B*	3	*D3S1578*	*D3S3658*	12.80	287	161	74
*USH2C*	5	*D5S428*	*D5S433*	18.54	269	34	13
*VRNI*	11	*INT2*	*D11S873*	22.41	311	89	37
*WFS2*	4	*D4S2366*	*D4S3023*	2.18	26	10	5
*WGN1*	5	*D5S626*	*D5S2103*	7.11	84	9	4

^a) ^See additional File 14; ^b) ^Linkage to a single DNA marker reported. An arbitrary locus size of 10.0 Mb was assigned.

## Discussion

Compiling the transcriptome of a cell or tissue is arguably more demanding than establishing the number of gene loci encoded by a given genome sequence [43]. This may mainly be explained by the dynamic nature of mRNA itself which frequently produces alternative transcripts from a single gene locus by usage of tissue-specific promoters, cryptic splice sites or variable polyadenylation signals [44,45]. In addition, variation in gene expression is known to occur within and between populations [46,47] and allele-specific expression, even from non-imprinted genes, appears to be common [48]. Further complicating transcriptome definition are effects of gender and age on RNA expression [49] as well as agonal and postmortem factors which greatly affect RNA integrity and thus frequently influence subsequent analyses [50]. Finally, differences in experimental technologies and data post-processing add an additional level of variability. Taken together, the complexities in mRNA metabolism and experimental data handling strongly suggest that there is not a single transcriptome for a given cell or tissue but implies an arbitrary number of individual transcriptomes which need to be defined by a series of parameters such as age, gender, ethnicity, cause and time of death of the tissue donor besides many others. It is therefore advisable to initially aim for a reference transcriptome providing a blueprint of an expression profile within a broadly defined time-frame. Following this line of reasoning, we here present a framework of a first reference transcriptome of the retina/RPE consisting of 13,037 unique transcripts which broadly characterize the mature state of expression in this tissue.

The present meta-analysis has integrated information from 27 studies employing diverse technologies to identify retinal/RPE transcrips. Among these, SAGE represents a sensitive tool to detect low level transcription [51] while the PCR-based SSH method is well suited to enrich for differentially expressed genes [36]. The combined use of these approaches together with conventional cDNA library sequencing and microarray-based techniques provides a more solid assessment of gene expression than would each method alone. For example, SAGE is based on sequencing of hundreds of thousands of short (10, 14, or 21 bp) tags, ideally derived from a unique location of a single transcript. Rare tags could originate from infrequently expressed transcripts but could also reflect minor genomic contamination or minor sequencing errors. For the assembly of the reference retinome we have addressed these concerns by including only those transcripts that have independently been confirmed in a second unrelated study. This has led to a conservative assembly of the 13K retinome. It should be kept in mind however that this proceeding likely excludes a number of authentic transcripts. This is illustrated by the finding that the 15K retinome which comprises 15,645 transcripts including those which were solely found in a single study (Table 2), contains an additional five of the 102 known retinal disease genes (*RHOK*, *MTATP6*, *CHM*, *LRAT*, *RIMS1*) not included in the 13K retinome. Similarly, an additional three genes (*RHOK*, *LRAT*, *GPRK7*) involved in the vitamin A/phototransduction pathway are part of the 15K but not the 13K retinome. With additional transcription data on the retina/RPE becoming available, a second generation retinome map will need to address this issue.

The estimation of transcriptome size represents one of the fundamental questions in molecular biology. Early studies using reassociation kinetics have calculated the number of distinct mRNA transcripts present in various mouse tissues to be between 11,500 and 12,500 [52]. Initial SAGE analyses have led to the conclusion that the number of different transcripts observed in normal and tumorous tissue may lie between 14,247 and 20,471 [53]. Recent data from comprehensive EST sequencing of a number of tissues including brain, breast, colon, head/neck, kidney lung, ovary, prostate, and uterus suggest expression of between 7,500 and 13,500 distinct genes for each tissue [54]. Although the size of the reference retinome is consistent with these estimates, the question of adequate transcript representation by the current compilation remains open. We have addressed this by defining a number of gene groups with known expression in retina/RPE and comparing these to the reference retinome. Genes exclusively expressed in retina/RPE are highly represented in the retinome (100%), as are mainly tissue-specific genes known to play a role in the vitamin A/phototransduction pathway (93%) (Table 3). A partial list of 260 genes whose encoded proteins were shown by immunohistochemistry to be expressed in the retina/RPE (but may also be present in other tissues), were represented in the reference retinome at a rate of approximately 79%. Similar numbers were obtained for the retinome coverage of retinal disease genes (85%). From these data we conclude that the 13K reference retinome is highly representative of retina/RPE-expressed genes and may describe as much as 90% of the transcript complement in the adult state.

Another point of interest concerns the proportion of retinome transcripts which is uniquely expressed in this tissue. Brentani et al. [54] estimate that any two tissues may share between 73% and 84% of their transcriptomes. Comparing transcription in three tissues (breast, colon, head/neck) the authors found overlapping expression in 47% of transcripts. To investigate this in more detail, we have compiled three partial transcriptomes from heart (n = 3,660), liver (n = 5,780) and prostate (n = 7,018) by applying the same stringent criteria as defined for the retinome. Limited by the size of the partial heart transcriptome, we determined 2,330 transcripts (termed "housekeeping" genes) to be expressed in all four tissues (i.e. 64% of the heart transcriptome). Comparing the retinome to *any *of the partial transcriptomes revealed overlapping gene profiles between 92 % and 95 %. This would suggest that only a minor proportion of retinome transcripts is indeed unique to the retina/RPE. Thus far, we have identified a group of so called "retinome-enriched" genes comprising 5,051 transcripts which are not present in the partial transcriptomes of heart, liver and prostate. This group most likely contains additional "housekeeping" or tissue-restricted transcripts and needs further adjustment by more refined *in-silico *normalization to comprehensive reference transcriptomes of other tissues.

Highly expressed genes including those with a ubiquitous or a tissue-specific transcription profile, have been shown to cluster in chromosomal regions of increased gene expression (termed RIDGEs) [55,56]. Functionally, this higher order structure has been related to transcriptional regulation [56,57]. To search for a possible correlation, we have determined the chromosomal distribution of the reference retinome independent of gene density. Our data show good agreement with the previously established regional expression map defining approximately 30 RIDGEs within the human genome. Overlaps are most evident for chromosomes 6, 9, 11, 17, and 19. From this we conclude that the majority of transcripts assembled in the reference retinome share characteristics of the RIDGEs including moderate to high level expression. This finding may be ascribed to the stringent selection criteria we have applied to assemble the reference retinome by excluding all transcripts (n = 2,608) that were reported in only a single study. Conversely, the RIDGE-like pattern of the reference retinome could be an indication that missing transcripts may have features compatible with chromosomal domains defined as anti-RIDGEs [56]. As opposed to RIDGEs, clustering of genes in anti-RIDGEs seems associated with significant decreased expression [56]. In contrast to their fractional occurrence in transcriptomes, the identification of such low abundant transcripts are likely to require significant resources in order to compile more complete transcriptomes.

To provide positional candidates for retinal disease genes, we have mapped the transcripts representing the reference retinome to the minimal regions defined for 42 retinal disease loci with as yet undefined gene mutations. To further limit the number of candidate genes, in particular for loosely defined disease loci such as *RP28 *or *VRNI*, we have similarly integrated the "retinome-enriched" transcripts. This also accommodates for the fact that approximately 50% of retinal disease genes are retina/RPE-specific [58]. For 41 of 42 unknown disease genes we have now identified strong candidates although for some disease loci including *AIED*, *COD4*, *CORD8*, *CRV*, *LCA5*, *RP28*, and *USH2B*, the number of candidates may still exceed capacities of most laboratories for direct analysis. For other disease loci (e.g. *BCD*, *BBS3*, *COD2*, *CYMD*, *MCDR4*, *OPA4*, *PRD*, *RNANC*, *RP24*, *RP29*, *RP6*, *WFS2 *and *WGN1*), a restricted number of candidates are now available (see additional File 14).

## Conclusions

We here present a first near-complete transcriptome of a defined tissue, the retinome, which may serve as a reference for further efforts to establish spatial, i.e. cell-specific, and developmental transcriptomes of the retina/RPE. A fundamental aspect of the current study was to integrate the available information on gene identification generated by a wide range of techniques. This ensures robustness and reliability of transcript data providing a stringent framework for further expression studies in systems biology. A similar approach for other tissues/cells would be advisable as this may greatly facilitate *in-silico *identification of tissue-specific genes to elucidate functional pathways vital for a defined cell population. In addition, the reference retinome may prove valuable for providing strong candidates for hereditary as well as genetically complex diseases and thus may help to further our understanding of retinal biology in health and disease.

## Methods

### Data retrieval and analysis

To assemble a list of genes expressed in the adult mammalian retina and RPE we reviewed 27 studies reporting raw or processed transcript data derived from several mammalian species including *H. sapiens*, *B. taurus*, *C. familiaris *and *M. musculus *(Table 1). The data were generated by cDNA library sequencing, microarray studies, and SAGE. Publically available data analysing transcripts from adult mammalian retina/RPE tissues published until December 2003 were included. Excluded were studies investigating transcription in retina/RPE by using RNA sources such as fetal tissues, cell lines or non-mammalian species. Gene identifiers such as GenBank accession number, gene nomenclature symbol, gene description, UniGene cluster ID, cDNA sequences or tags were available from sources as detailed in additional File 15 and were used to retrieve the unique human LocusLink ID for each gene (as of December, 2003). Only genes with established LocusLink ID were included in the present study. For SAGE data, tag-to-gene assignment was done by querying the SAGEmap_tag_ug-rel dataset [59,60]. Tags assigned to multiple genes were excluded from further analysis. Human orthologous genes were established via the NCBI-curated homology database[61] or by BLAST sequence comparison [62].

To assemble partial transcriptomes of heart, liver and prostate, for each tissue data were mined from at least one SAGE library, in addition to expressed sequence tag (EST) sources (see additional File 16). Similar to the criteria for the assembly of the retinome, genes identified in only one study were disregarded. EST retrieval was facilitated by use of the Gene Library Summarizer [63] which retrieves the known genes represented by at least one EST and generated from a tissue sample with normal histology.

Partial lists of genes known to play a role in the retina and/or the RPE were assembled from the literature (see additional Files 5, 6, 7 and 8). Additional File 5 summarizes genes known to be exclusively expressed in retina and/or RPE, while additional File 6 includes genes involved in the phototransduction cascade and the vitamin A cycle. Additional File 7 is a partial compilation of genes/proteins verified by immunohistochemistry to be present in adult mammalian retina and/or RPE. A list of 102 genes involved in retinal diseases was retrieved from the RetNet database, January 2004 [58] (see additional File 8).

### Assignment of genes and disease loci to the human genome

A total of 43,109 human non-redundant syntenic gene predictions (SGP) were retrieved (as of December 2003) and chromosomally mapped to the reference sequence of the human genome (July 2003) utilizing the USCS Genome Table Browser [64]. Based on the position of their putative transcription start sites, the SGPs were assigned to 5 Mb bins along the human chromosomes. In addition, one-megabase bins were defined for refined analysis of chromosome 6 and 19 (Fig. 1b). Similarly, the chromosomal map positions of the retinome transcripts were determined by querying the USCS Genome Table Browser with the respective LocusLink, UniGene or RefSeq IDs.

Mapped loci of retinal dystrophies with unknown genetic basis (n = 45) were taken from RetNet, January 2004 [58] and placed on the human genome sequence by querying the USCS Genome Table Browser with DNA marker sequences shown to flank the minimal candidate region. Three disease loci (*CORD1*, *CORD4 *and *RCD1*) are insufficiently mapped on the respective human chromosomes and were therefore not included in the analysis.

### Statistical analysis of gene distribution

To determine if either of the two datasets, the 43,109 human non-redundant SGPs and the 13K retinome transcripts, is distributed in a non-parametric and distribution free manner over the genome, the Kolmogorov-Smirnov Goodness-of-Fit Test was used [65]. Statistical significance of the median difference in paired chromosomal distribution of retinome transcripts versus the SGPs was then evaluated by the non-parametric Wilcoxon two-sample paired signed rank test [66]. To carry out the test we calculated the difference between all genes versus retinal genes per 5-Mb bin. To correct for the total number of genes within the two groups, the SGPs per bin were adjusted by a factor of 13,037/43,109 = 0.30. Mean and median values per bin were 21.05 and 10.93 for all genes and 21.26 and 19.93 for retinal genes, respectively.

## Authors' contributions

HLS participated in the design of the study, collected and processed the information from the published reports related to the retina and RPE genes. TG conceptually developed the RetinaCentral database and is involved in the curation of the site. JK carried out the statistical analyses and helped with the computational handling of data. BHFW was involved in all aspects of data assembly and prepared the manuscript. All authors read and approved the final manuscript.

## Supplementary Material

Additional File 1 **Retina/RPE genes reported only in a single study **List of genes expressed in the retina or RPE reported only in one study Click here for file

Additional File 2 **Gene list of the reference retina / RPE transcriptome **List of 13,037 genes expressed in the retina/RPE Click here for file

Additional File 3 **List of genes identified exclusively in retina studies** Click here for file

Additional File 4 **List of genes identified exclusively in RPE studies** Click here for file

Additional File 5 **Partial list of genes known to be expressed specifically in the retina and/or RPE** Click here for file

Additional File 6 **Partial list of genes known to be involved in the vitamin A cycle and phototranduction pathway** Click here for file

Additional File 7 **Partial list of genes known to encode retina / RPE proteins** Click here for file

Additional File 8 **List of known genes involved in retinal diseases** Click here for file

Additional File 9 **Partial gene list of the heart transcriptome** Click here for file

Additional File 10 **Partial gene list of the liver transcriptome** Click here for file

Additional File 11 **Partial gene list of the prostate transcriptome** Click here for file

Additional File 12 **List of genes present in the reference retinome and partial transcriptomes of heart, liver, prostate** Click here for file

Additional File 13 **List of genes present in partial transcriptomes of heart, liver, and prostate but not in reference retinome** Click here for file

Additional File 14 **List of retinome transcripts mapping to retinal disease intervals.** Click here for file

Additional File 15 **Data sources used to compile the retina / RPE transcriptome** Click here for file

Additional File 16 **Data sources used to compile partial transcriptomes of heart, liver, and prostate** Click here for file
